# Gone but Not Forgotten: Ovarian Metastasis From a Colon Carcinoma in a 19-Year-Old Female

**DOI:** 10.7759/cureus.9466

**Published:** 2020-07-29

**Authors:** Talal Almas, Muneeb Ullah, Mehwish Kaneez, Syed Muhammad Jawad Zaidi, Muhammad Kashif Khan

**Affiliations:** 1 Internal Medicine, Royal College of Surgeons in Ireland, Dublin, IRL; 2 General Surgery, Maroof International Hospital, Islamabad , PAK; 3 Internal Medicine, Rawalpindi Medical University, Rawalpindi, PAK; 4 Surgical Oncology, Federal Government Poly Clinic (Post Graduate Medical Institute), Islamabad, PAK; 5 Surgical Oncology, Maroof International Hospital, Islamabad, PAK

**Keywords:** giant ovarian tumors, metastatic ovarian cancer, colon cancer

## Abstract

Ovarian tumors occurring secondarily to metastatic colorectal carcinoma remain a rare occurrence. Since ovarian tumors remain predominantly asymptomatic in the initial stages, they are often diagnosed incidentally. The vague, non-specific symptoms elicited by a secondary ovarian carcinoma, coupled with a histopathology remarkably similar to that evoked by primary ovarian tumors, render its ascertainment a diagnostic challenge. We hereby delineate an interesting case of a metachronous ovarian adenocarcinoma in a 19-year-old patient with a prior medical history significant for colorectal carcinoma treated with hemicolectomy. Subsequent diagnostic workup divulged a mass in her left adnexal region, which was ultimately diagnosed as a metastatic colon adenocarcinoma to the ovary. Unfortunately, the patient succumbed to the aggressive malignancy and did not survive. We therefore aim to accentuate the diagnostic and therapeutic dilemmas fomented by ovarian adenocarcinomas that arise secondarily to primary colorectal cancers.

## Introduction

Ovarian carcinomas encompass a vast array of cancers. Primary ovarian carcinomas are indigenous tumors that arise from the ovarian parenchyma. Secondary ovarian cancers, on the other hand, are exceedingly diverse and may arise from a primary metastatic lesion afflicting an extra-ovarian region in the body. An estimated 5%-30% of all ovarian malignancies occur due to metastases from other organs, with more than half of these occurring bilaterally [1]. The common primary sites from where metastases to the ovaries ensue are the gastrointestinal tract (stomach, appendix, and colon), breast, pancreas, uterus, and contralateral ovary [2]. Of these metastatic tumors, roughly 50%-90% occur due to metastatic proliferation of a combined gastrointestinal tract and breast primary cancer. Nevertheless, metastatic colon adenocarcinoma to the ovary remains an exceedingly rare etiology and accounts for merely 3%-8% of the cases, purporting a meagre prevalence of 1.1% [3].

Secondary ovarian cancers predominantly present synchronously but can rarely manifest metachronously as well. A metasynchronous ovarian carcinoma secondary to an underlying colon cancer has been observed in merely 5% of the cases reported [4]. Metastasis to the ovaries from extragenital sites can occur through the means of direct extension from adjacent organs or may involve lymphatics, blood vessels, or transcoelomic extension from abdominal cancers, culminating in the abnormal growth impaling on the ovaries [5]. In a plethora of cases, the patient may have a history of the primary tumor; however, in a majority of the cases, the patients may present with vague, non-specific symptoms, such as pain or abdominal distention, elicited by an ovarian mass [6]. In such instances, secondary ovarian tumors are erroneously diagnosed as primary ovarian cancers due to the remarkable resemblance in histopathological features of the metastatic and primary tumors, resulting in diagnostic misinterpretation, ineffective management, and thus portending a worse prognosis [7]. The metastatic spread of colon carcinoma to the ovaries, due to its ambiguous mode of spread and tumor behavior, therefore poses a perplexing diagnostic dilemma for the clinicians. This study elucidates the case of a rare metachronous ovarian tumor that arose secondarily to a primary colorectal carcinoma in a 19-year-old female.

## Case presentation

**Figure 1:**
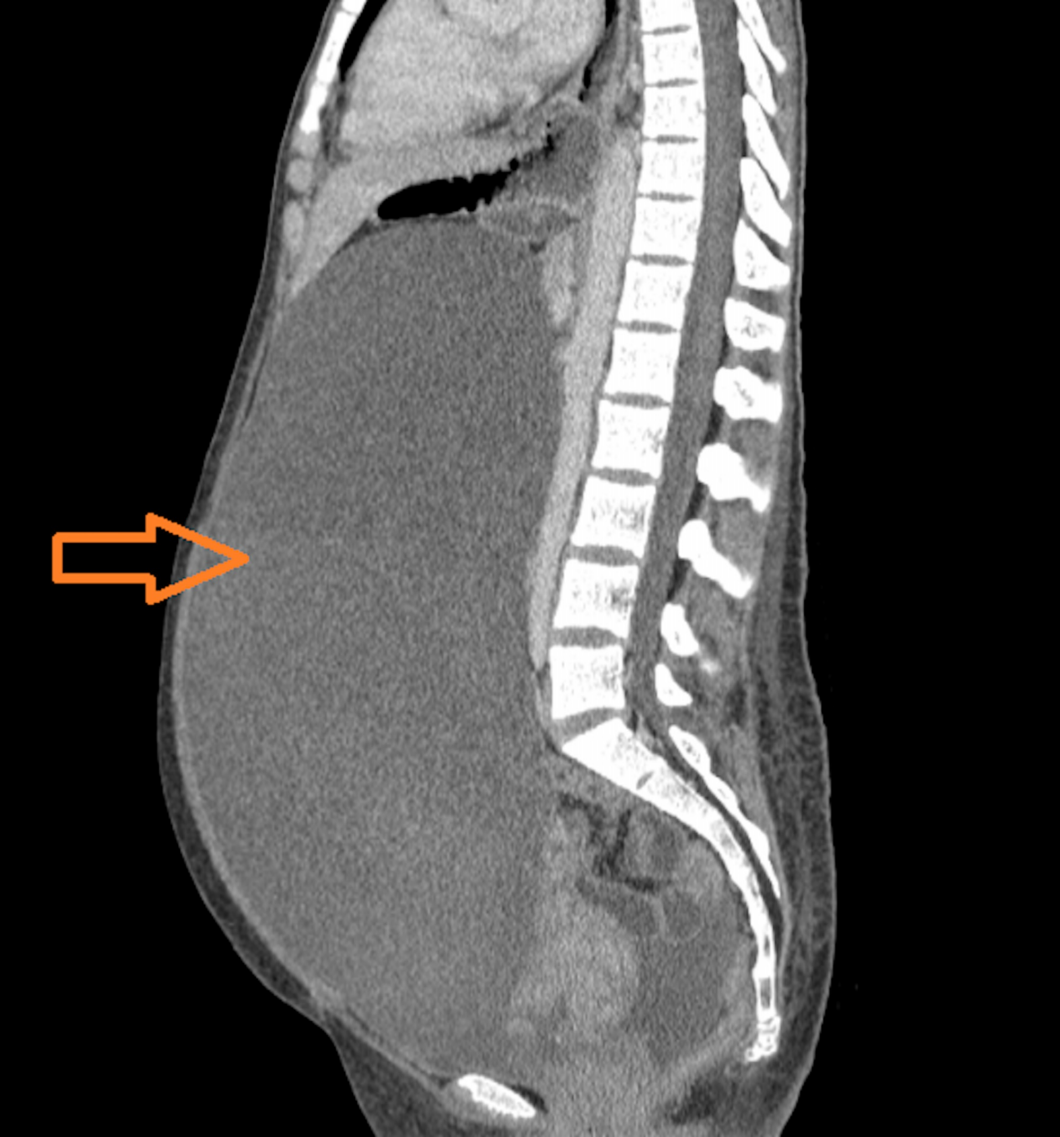
A sagittal CT scan (abdomen and pelvis) depicting an exorbitant mass (arrow) compressing the surrounding abdominal structures.

We delineate the case of a 19-year-old female with no known comorbid conditions. The patient’s prior surgical history was significant for an appendectomy performed two years ago. The resected appendix sample was sent for histopathology, which subsequently revealed a mucinous adenocarcinoma afflicting the appendix. The patient was then advised to undergo a right hemicolectomy, which was performed shortly thereafter without any complications. Histopathological analysis of the excised colon specimen revealed an underlying colon adenocarcinoma. Following the hemicolectomy, the patient was referred to a medical oncologist and received six cycles of chemotherapy. The patient then got married and subsequently became pregnant. During the last trimester of her pregnancy, the consultant gynecologist adeptly identified an unsual mass in the patient’s left adnexal region. The patient was thus advised to follow up with the gynecologist for the evaluation of the aforesaid mass after her pregnancy. Thereafter, the patient gave birth to a premature baby who passed away 10 days after birth. Left to convalesce and recuperate from the tragic loss, the patient observed that the left adnexal mass that the gynecologist had previously identified grew exorbitantly. The patient then presented to our department with a history of dyspnea, lower limb edema, abdominal pain, an inability to tolerate solid foods, and increasing constipation. A CT scan of the chest, abdomen, and pelvis was thus ordered and divulged a mass measuring 26.5 x 14.7 x 34 cm in the abdomen that appeared to arise from the left adnexal region (Figure 1). 

**Figure 2:**
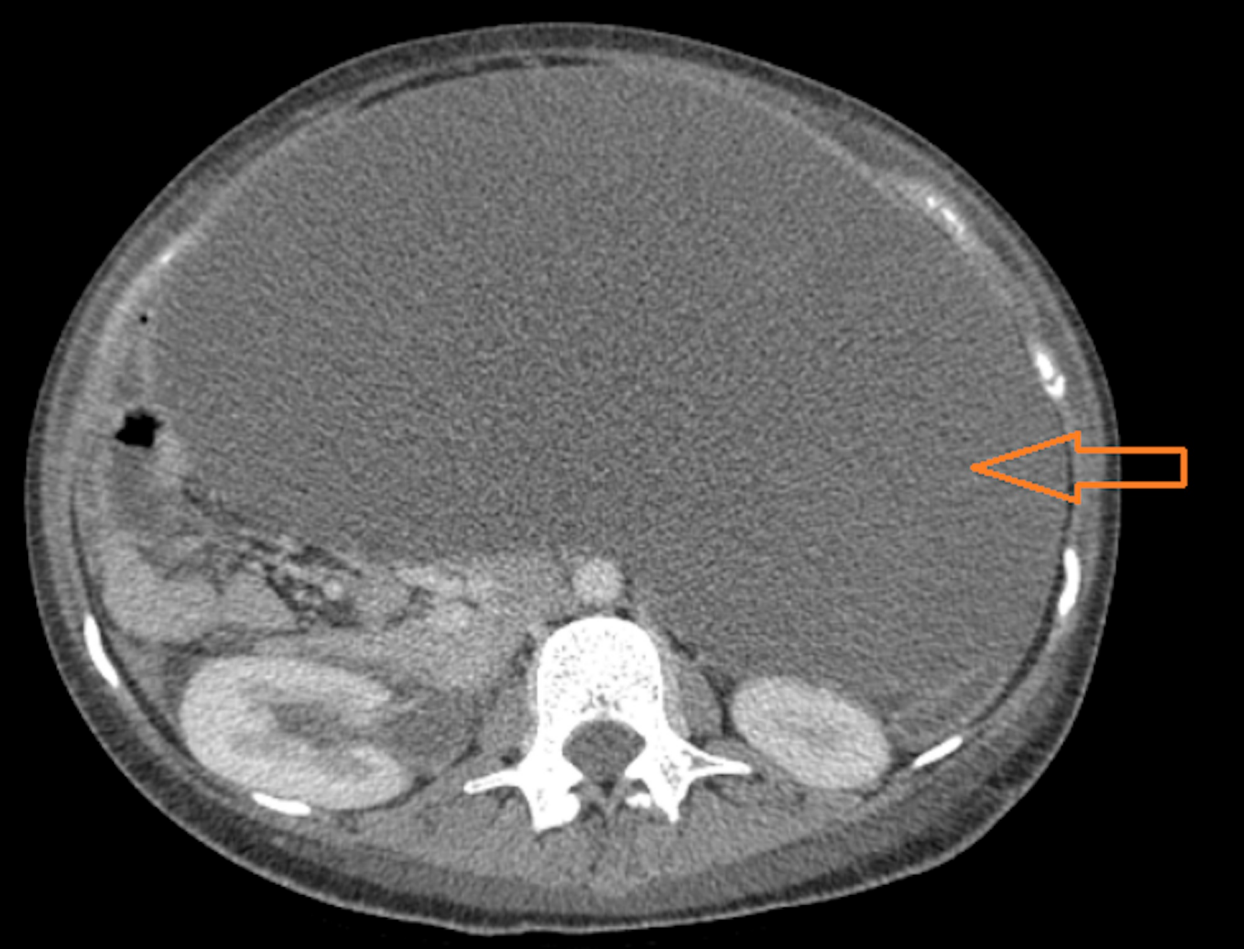
An axial CT scan (abdomen and pelvis) delineating an exorbitant mass (arrow) occupying the entire abdomen and pelvis.

Furthermore, the abnormal growth was noted to span across the entire abdominal region (Figure 2).

**Figure 3:**
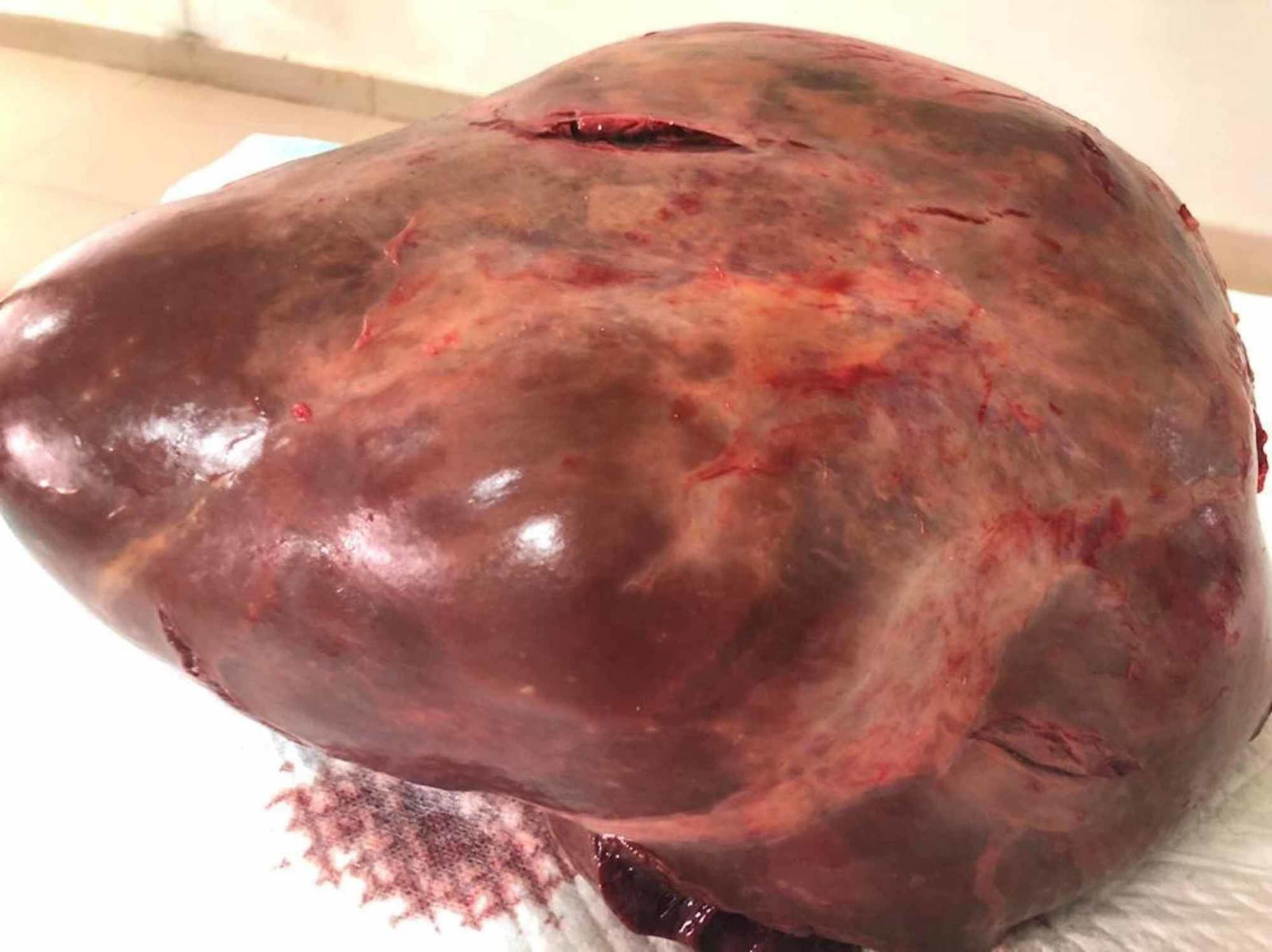
The gross morphology of the excised specimen.

The case was then discussed in the multidisciplinary team (MDT) meeting, and a recommendation vouching for the surgical excision of the mass was made. Preoperatively, the patient was commenced on anticoagulation therapy. A left radical salpingo-oophorectomy along with omentectomy was performed, yielding the specimen elucidated in Figure 3. 

Peritoneal sampling and biopsy of the lesion afflicting the right ovary were simultaneously performed. The patient’s postoperative recovery was uneventful, with the patient experiencing an immediate relief of her symptoms. Histopathological evaluation of the sample divulged a metastatic colon adenocarcinoma to the ovary. The patient’s complex etiology was discussed in an MDT meeting, and the patient was subsequently initiated on chemotherapy. Following chemotherapy, the patient remained disease free for six months and was on regular follow-up. However, after six months, the disease was noted to have disseminated widely. The patient was therefore advised to undergo palliative treatment; however, the patient did not survive, succumbing to her widespread malignancy. 

## Discussion

Colon carcinoma has been noted to commonly spread to the liver and the peritoneum while reports of its metastasis to the ovaries remain sparse. When it does occur, metastasis to the ovary from a primary colon cancer portends a relatively poor prognosis. In such instances, the median survival time hovers around nine months [5]. Colorectal carcinoma is predominantly a disease of the elderly, with more than half of the carcinomas diagnosed after the age of 70 years. Merely 10% of the cases of colorectal carcinoma are diagnosed before the age of 55 years [8]. Screening for the high-risk subtype of colorectal carcinoma is usually performed between 50 and 75 years of age [9]. Given this aforementioned age distribution, the presence of colon carcinoma in a 19-year-old patient is an exceedingly rare entity. Furthermore, the presence of metastatic colon adenocarcinoma to the ovary, as observed in our case, is even rarer [8,9]. 

Secondary ovarian carcinomas are noted to boast a significant morbidity and present with non-specific symptoms, such as abdominal pain, distention, constipation, and anemia [4,6]. However, most of the patients remain asymptomatic, an aspect that further obscures the timely diagnosis of a secondary ovarian carcinoma. Supplanting the vague constellation of symptoms elicited by ovarian tumors is the notion that it is also difficult to discriminate between a primary and secondary ovarian malignancy because of the exceedingly similar morphological findings and clinical course [3]. It had previously been believed that bilateralism and a solid nature associated with a metastatic mass may help in discriminating primary ovarian tumors from the secondary ones [2]. However, a subsequent study concluded that these aforesaid features are not exclusively suggestive of secondary ovarian disease since solid masses can gradually morph into cystic masses due to extensive necrosis, further complicating the ascertainment of an apt clinical diagnosis [10]. Immunohistochemical investigations, in the aftermath of tumor resection, can therefore help in establishing the true etiology of the underlying malignancy. 

In our case, a left radical salpingo-oophorectomy was performed to excise the ovarian mass. Additionally, an omentectomy, along with peritoneal fluid cytology and biopsy of the lesion in the contralateral ovary, was performed. Thereafter, the patient was commenced on a chemotherapy regimen. Interestingly, it has been reported that metastatic colon adenocarcinomas to the ovary are usually resistant to chemotherapy [1,11]. However, due to sparse data and unavailability of proper guidelines, the risk stratification of patients for surgery or palliative care remains an onerous undertaking. There is thus an unmet need for the curation of specific guidelines that would better inform the debate pertaining to the optimal treatment regimen in such instances. 

## Conclusions

Secondary ovarian carcinomas are frequently misdiagnosed as primary ovarian cancers, leading to the subsequent mismanagement of the afflicted patient and yielding a worse prognosis. Secondary ovarian malignancy therefore merits a place in the list of differential diagnosis pertaining to any abnormal adnexal masses. Furthermore, an unusually young age at presentation should not preclude the diagnosis of either a primary or a secondary malignancy afflicting the ovaries. An MDT approach remains pivotal in ascertaining the true underlying diagnosis and in devising optimal treatment strategies.
